# An Indicator contribution-oriented assessment framework for identifying dominant fire risk drivers in cable tunnels: Development and case study

**DOI:** 10.1371/journal.pone.0348198

**Published:** 2026-05-05

**Authors:** Liming Li, Yongyu Wang, Bo Liu, Liwei Song, Wentao Ma

**Affiliations:** 1 Shenyang Fire Science and Technology Research Institute of MEM, Shenyang, Liaoning, P.R. China; 2 Liaoning Key Laboratory of Fire Prevention Technology, Shenyang, Liaoning, P.R. China; China Construction Fourth Engineering Division Corp. Ltd, CHINA

## Abstract

Cable tunnels are critical components of urban power supply systems, where fire incidents can cause severe safety consequences due to high cable density and confined environments. Effective fire risk management is challenged not only by risk quantification, but also by limited understanding of which factors dominantly drive fire risk variation across tunnel segments.This study proposes a contribution-oriented fire risk assessment framework for cable tunnels that integrates hierarchical indicator weighting with indicator contribution analysis. A structured fire risk indicator system is established based on fire accident mechanisms, engineering standards, and expert knowledge. A composite fire risk index is calculated for segmented tunnel units, and a perturbation-based contribution analysis is conducted to quantify the marginal influence of individual indicators on overall risk levels. Scenario-based analyses are further employed to examine the effectiveness of alternative fire prevention and management strategies. A case study shows that cable tunnel fire risk is governed by a limited number of dominant drivers rather than uniform contributions from all indicators. Structural integrity–related factors, such as fire door integrity and joint defects, together with key operational and management indicators, exhibit the strongest influence on fire risk formation. The results also reveal pronounced spatial heterogeneity in fire risk distribution and differentiated responses to management interventions. By identifying dominant fire risk drivers and their contribution characteristics, the proposed framework enhances the interpretability and practical value of fire risk assessment, supporting targeted risk control and resource prioritization in cable tunnel safety management.

## 1. Introduction

With the continuous expansion of urban underground space and the increasing reliance on centralized power supply systems, cable tunnels have become an indispensable component of modern infrastructure. By accommodating large quantities of power cables within confined underground environments, cable tunnels improve spatial utilization and operational efficiency. However, this structural advantage is inherently accompanied by elevated fire risk, as combustible cable insulation materials, dense cable layouts, and restricted ventilation conditions jointly facilitate rapid fire growth once ignition occurs [1–3]. From a broader perspective, cable tunnels represent a critical class of urban underground infrastructure whose vulnerability under extreme events may lead to disproportionate societal and economic consequences [4,5].

Cable tunnel fires are typically characterized by strong concealment, rapid propagation, and high difficulty in emergency response. Beyond direct fire damage, such incidents may result in prolonged power outages and trigger cascading failures in interconnected urban or industrial systems [6–8]. For cable tunnels serving critical facilities or industrial zones, fire-induced service interruptions can severely compromise operational continuity and safety management, highlighting the necessity of systematic fire risk evaluation and effective preventive strategies [9].

Over the past decades, a wide range of approaches has been developed to assess fire risk in cable tunnels and other underground infrastructures, including qualitative judgment methods, semi-quantitative scoring models, and quantitative probabilistic techniques [10–12]. These approaches generally involve the identification of relevant risk factors, estimation of their likelihood, and evaluation of potential consequences. Quantitative methods such as fuzzy comprehensive evaluation, Bayesian networks, and probabilistic graphical models have demonstrated effectiveness in representing complex fire risk structures and supporting safety-related decision-making [13–16]. Nevertheless, many quantitative approaches rely heavily on detailed statistical data or complex parameter calibration, which may limit their applicability in routine engineering practice, particularly when historical data are sparse or operational conditions vary significantly across facilities [17–19].

Multi-criteria decision-making methods have attracted increasing attention for fire risk assessment due to their ability to integrate heterogeneous risk factors and expert knowledge within a unified analytical framework. Among these methods, the Analytic Hierarchy Process (AHP) has been widely applied in fire safety and infrastructure risk studies because of its transparent hierarchical structure and systematic weighting mechanism [20–22]. By decomposing complex systems into multiple indicator levels, AHP enables structured evaluation of the relative importance among risk factors and provides an intuitive basis for comprehensive risk aggregation.

However, existing AHP-based fire risk assessment studies have predominantly focused on indicator weight determination and overall risk ranking, while the contribution of individual indicators to risk variation has rarely been examined in depth [23,24]. From a practical risk management perspective, decision-makers are often more concerned with identifying which factors dominantly drive risk formation and how targeted interventions can most effectively reduce overall risk levels. Without contribution-oriented analysis, conventional risk assessment outcomes may be insufficient to support refined management strategies or efficient allocation of limited safety resources [25–27].

To address this gap, the present study proposes a contribution-oriented fire risk assessment framework for cable tunnels that integrates AHP-based indicator weighting with indicator contribution analysis. A hierarchical fire risk indicator system is established to capture structural conditions, operational states, environmental characteristics, and management measures. Based on AHP, indicator weights are derived and used to calculate a composite fire risk index for segmented tunnel units. Furthermore, a perturbation-based contribution analysis is conducted to quantify the influence of individual indicators on overall risk variation, and scenario-based comparisons are employed to examine the effectiveness of alternative risk control strategies. The proposed framework aims to enhance the interpretability and practical value of fire risk assessment outcomes, thereby supporting risk-informed decision-making in cable tunnel fire safety management.

## 2. Method

### 2.1. Overall framework of the proposed fire risk assessment model

To systematically assess fire risk in cable tunnels and quantify the contribution of individual risk indicators, a contribution-oriented assessment framework based on the AHP is developed. The proposed framework consists of four sequential stages:

a) Construction of a hierarchical fire risk indicator system.
b) Determination of indicator weights using AHP.
c) Calculation of a comprehensive fire risk index for individual tunnel segments.
d) Indicator contribution analysis combined with scenario-based risk evaluation.

**Fig 1:**
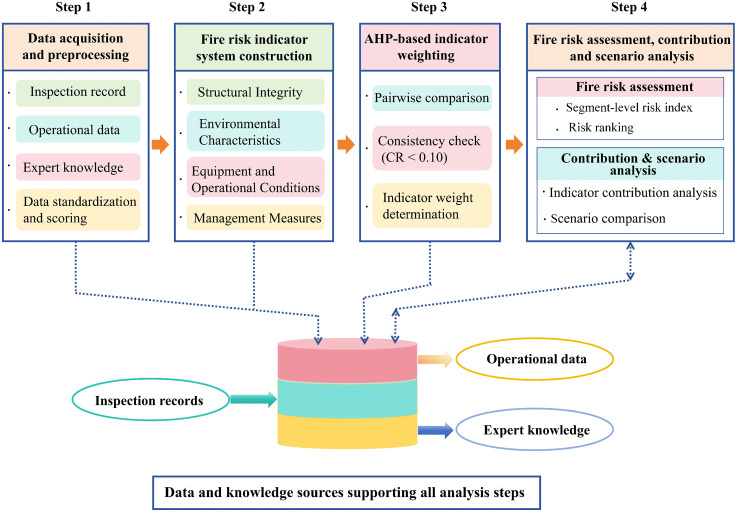
Overall framework of the proposed cable tunnel fire risk evaluation model.

The framework is designed to ensure methodological transparency and reproducibility by clearly separating indicator weighting, risk aggregation, and contribution analysis. The logical relationships among these stages are illustrated in Fig 1, which provides an overview of the entire methodological workflow.

### 2.2. Construction of the fire risk indicator system

A hierarchical fire risk indicator system was constructed based on three complementary sources: fire accident mechanisms, engineering standards, and previous research on underground infrastructure fire safety. Based on these principles, the fire risk indicator system was organized into three hierarchical levels: the target level, the criterion level, and the indicator level. The principles are explained as follows:

First, from the perspective of fire accident mechanisms, the indicator system aims to capture the key processes governing cable tunnel fire formation and development, including ignition potential, fire propagation pathways, smoke and heat accumulation, and emergency response capability. These mechanisms represent the fundamental physical and operational processes that determine fire risk evolution in confined underground environments and have been widely discussed in previous studies on tunnel fire behavior and underground infrastructure safety [1,2].

Second, relevant engineering standards and fire safety guidelines for power infrastructure and underground utility tunnels were considered during the indicator selection process. In particular, recommendations from fire protection standards such as NFPA 850 and commonly adopted engineering safety practices were referenced to identify critical structural and operational factors influencing cable tunnel fire safety.

Third, the indicator system was informed by previous studies on fire risk assessment in underground infrastructures and utility tunnels, which emphasize the importance of integrating structural conditions, operational states, environmental characteristics, and management measures within a unified evaluation framework [6,8].

The criterion level consists of four dimensions: structural integrity, equipment and operational conditions, environmental characteristics, and management measures, which jointly reflect the key mechanisms governing fire occurrence, development, and control in cable tunnels. Among them, structural integrity focuses on the inherent fire resistance of tunnel structures, while equipment and operational conditions characterize fire-related facilities and operational states. Environmental characteristics describe the physical and thermal environment in which fires may initiate and evolve, including factors related to ventilation, combustible accumulation, and the perception of fire-induced environmental changes. Management measures represent organizational and procedural factors that influence fire prevention and emergency response effectiveness.

The indicator level comprises specific risk factors that are observable or assessable in engineering practice, such as fire door integrity, joint defects, ventilation effectiveness, combustible dust accumulation, inspection frequency, and emergency drill readiness. All indicators are selected to avoid redundancy while ensuring comprehensive coverage of both inherent physical risk sources and management-related influences.

**Fig 2:**
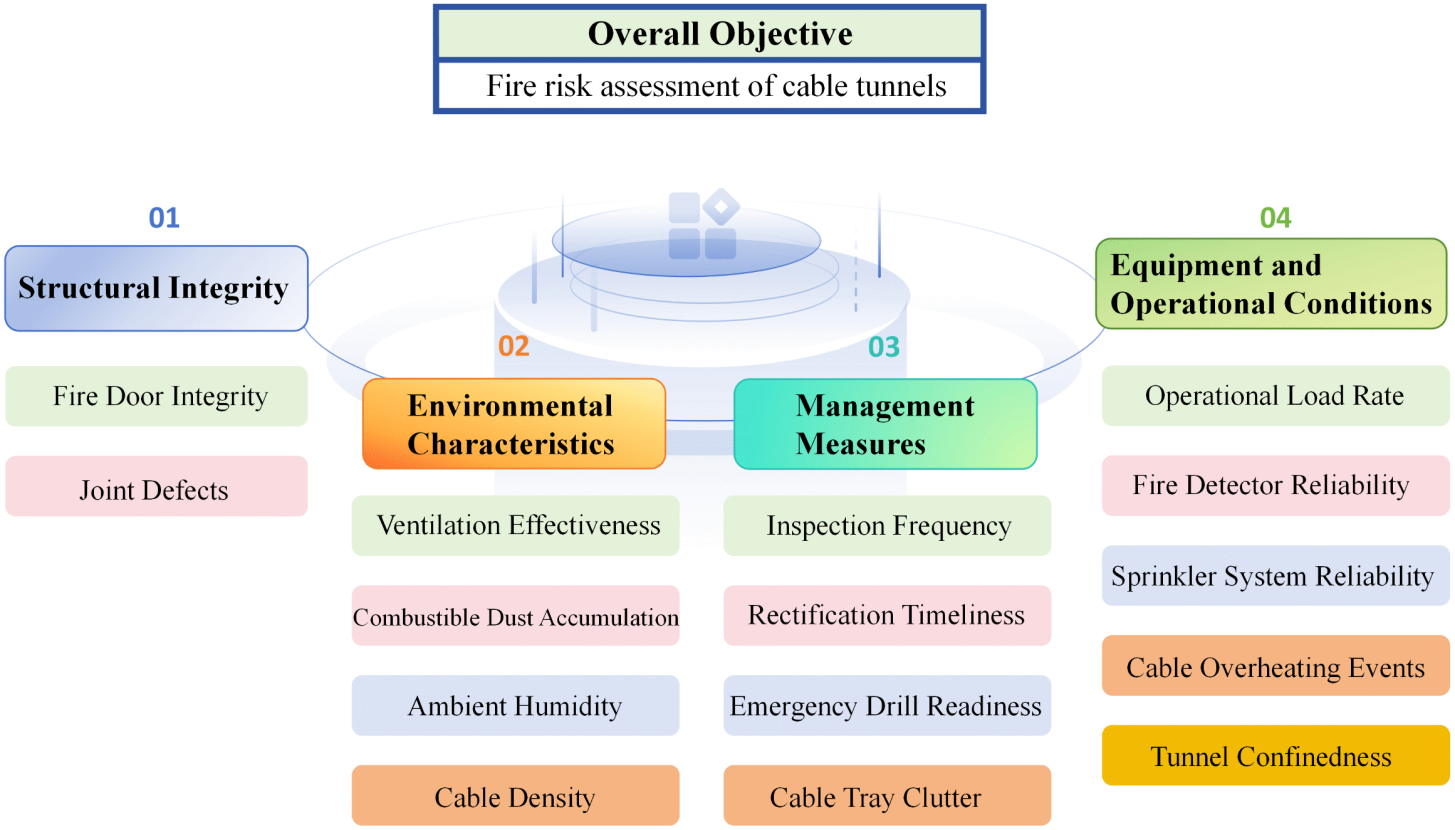
Hierarchical fire risk indicator system for cable tunnels.

The hierarchical structure of the fire risk indicator system is illustrated in Fig 2. For methodological completeness, the full set of indicators illustrated in Fig 2 is retained in the AHP weighting stage. Potential indicator dominance and overlapping effects are subsequently examined through indicator weighting and contribution analysis, rather than by excluding indicators a priori.

### 2.3. Determination of indicator weights using AHP

#### 2.3.1. Expert judgment and pairwise comparison.

The relative importance of the fire risk indicators was determined using AHP, a widely used multi-criteria decision-making method for complex engineering systems. The AHP approach enables structured comparison among indicators and provides a systematic framework for deriving quantitative weights based on expert knowledge.

A panel of nine experts participated in the evaluation process. The expert group consisted of three fire safety engineers, three cable tunnel operation engineers, and three infrastructure safety management specialists, each with more than ten years of professional experience in fire protection engineering, power infrastructure operation, or industrial safety management. The inclusion of experts from multiple professional domains ensured that the evaluation reflected both engineering and operational perspectives of cable tunnel fire risk.

Each expert independently constructed pairwise comparison matrices for indicators within the same hierarchical level using the standard nine-point AHP scale, where 1 indicates equal importance and 9 indicates extreme importance of one indicator relative to another. Reciprocal values were assigned for inverse comparisons. To obtain a group judgment matrix, the individual expert assessments were aggregated using the geometric mean method, which is commonly adopted in group decision-making AHP applications. The aggregated comparison elements $a_{ij}$ between indicators *i* and j is calculated as Equation (1).


$$\begin{array}{c} a_{ij}={\left(\prod_{k=\text{1}}^{n}\overset{\left(k\right)}{\underset{ij}{a}}\right)}^{\text{1}/n} \end{array}$$ (1)

where $\overset{\left(k\right)}{\underset{ij}{a}}$ denotes the pairwise comparison value between indicators *i* and j provided by expert *k*, *n* represents the number of experts, $a_{ij}$ is the aggregated comparison value used to construct the final group judgment matrix. For transparency and reproducibility, the complete set of AHP pairwise comparison matrices used in this study is provided in S1 Appendix.

#### 2.3.2. Weight calculation and consistency verification.

For each judgment matrix, the corresponding indicator weight vector is derived from the normalized principal eigenvector. To ensure the reliability and internal logical coherence of expert judgments, consistency verification is conducted for all judgment matrices.The consistency ratio (CR) is calculated as Equation (2).


$$CR=\frac{CI}{RI}$$ (2)

where *CI* is the consistency index and *RI* is the random index.

A judgment matrix is considered acceptable if CR < 0.10, which is the commonly adopted threshold for acceptable consistency in AHP-based decision analysis. All judgment matrices constructed in this study satisfy the consistency requirement, indicating acceptable agreement among expert evaluations. The final indicator weights are obtained by synthesizing weights across all hierarchical levels.

#### 2.3.3. Example of expert judgment matrix.

To enhance the transparency and reproducibility of the indicator weighting procedure, an illustrative example of the expert judgment matrix constructed using AHP is provided in this subsection.

Table 1 presents an example of a pairwise comparison matrix constructed at the criterion level. The matrix illustrates how experts compare the relative importance of the major indicator groups defined in the hierarchical evaluation framework. In this illustrative matrix, a larger numerical value assigned to the comparison between two indicator groups indicates that one group is judged to have a stronger influence on cable tunnel fire risk than the other. Conversely, reciprocal values represent inverse judgments. For each judgment matrix, the weight vector is derived from the normalized principal eigenvector, and consistency verification is conducted to ensure the logical coherence of expert assessments.

**Table 1:** Example of pairwise comparison matrix for criterion-level indicators.

Criterion	Structural Integrity	Environmental Characteristics	Equipment and Operational Conditions	Management Measures
Structural Integrity	1	3	5	7
Environmental Characteristics	1/3	1	3	5
Equipment and Operational Conditions	1/5	1/3	1	3
Management Measures	1/7	1/5	1/3	1

It should be emphasized that the matrix shown in Table 1 is provided for illustrative purposes only. The actual indicator weights reported in this study are obtained by aggregating expert judgments across all relevant indicators and hierarchical levels. The final weighting results are summarized in Table 3.

### 2.4. Indicator scoring and comprehensive fire risk index calculation

#### 2.4.1. Indicator scoring criteria.

Each tunnel segment is evaluated according to the established fire risk indicator system. Indicator scores are assigned based on inspection records, operational data, and expert judgment. To ensure consistency and comparability across different indicators, a unified ordinal scoring scale is adopted for all indicator-level factors.

The indicator scores used in this study were derived from routine inspection records, operational monitoring data, and engineering assessments conducted for the studied cable tunnel system. The inspection records include periodic structural inspections, equipment maintenance logs, and safety management documentation collected during regular tunnel operation. These inspection records were obtained from routine tunnel inspection and maintenance activities conducted at regular operational intervals. For indicators that can be directly observed or measured, such as structural condition and equipment reliability, scores were primarily determined based on inspection and maintenance records. For management-related indicators, including inspection frequency and rectification timeliness, the scoring was derived from administrative maintenance records and standardized evaluation criteria established by the expert panel. This combination of inspection data and expert interpretation ensures both practical relevance and methodological consistency in the scoring process.

The inspection and operational data used for indicator scoring were collected from routine tunnel maintenance records covering approximately one year of operation. Routine safety inspections are typically conducted on a monthly basis, while operational parameters such as equipment condition and ventilation status are monitored continuously through the tunnel management system.

The representative scoring criteria for the indicator-level fire risk factors, derived from inspection records, operational data, and engineering evaluation standards, are summarized in Table 2, in which higher scores correspond to higher levels of fire risk. As shown in the table, all indicators are evaluated using the same three-level ordinal scale (1-3-5), while the specific qualitative descriptions are adapted to reflect the physical meaning and engineering characteristics of each indicator. Table 2 provides a standardized illustration of the scoring logic applied to the indicator-level fire risk factors. Although the qualitative descriptions differ among indicators, the underlying ordinal scale remains consistent to ensure comparability across different indicators. To avoid redundancy, separate scoring tables for individual indicators are not repeatedly presented. Detailed indicator definitions and extended scoring interpretations are provided in S2 Appendix.

**Table 2:** Representative scoring criteria for major fire risk indicators.

Indicator group	Indicator	Low risk (1)	Medium risk (3)	High risk (5)
Structural Integrity	Fire Door Integrity	Intact	Partially degraded	Failed
	Joint Defects	None	Minor defects	Severe defects
Equipment and Operational Conditions	Operational Load Rate	Light	High	Overloaded
	Fire Detector Reliability	Reliable	Degraded	Unreliable
	Sprinkler System Reliability	Reliable	Degraded	Unreliable
	Cable Overheating Events	Rare	Occasional	Frequent
	Tunnel Confinedness	Low	Moderate	High
Environmental Characteristics	Ventilation Effectiveness	Favorable	Restricted	Adverse
	Combustible Dust Accumulation	Minimal	Moderate	Severe
	Cable Density	Low	Moderate	High
	Ambient Humidity	Suitable	Fluctuating	Extreme
Management Measures	Inspection Frequency	Regular	Occasional	Insufficient
	Rectification Timeliness	Timely	Delayed	Severely delayed
	Emergency Drill Readiness	Regular	Occasional	Rare/None
	Cable Tray Clutter	Organized	Moderate	Severe

#### 2.4.2. Comprehensive fire risk index calculation.

To eliminate dimensional differences among indicators, all indicator scores are normalized prior to aggregation. The comprehensive fire risk index of tunnel segment j is calculated using a weighted summation model as Equation (3).


$$R_{j}=\sum_{i=\text{1}}^{n}w_{i}\cdot s_{ij}$$ (3)

where $R_{j}$ denotes the comprehensive fire risk index,${\;w}_{i}$ represents the weight of indicator i, and $s_{ij}$ is the normalized score of indicator i for segment j. The calculated risk indices are subsequently used for risk ranking and comparative analysis across tunnel segments.

### 2.5. Indicator contribution analysis

To quantify the influence of individual indicators on overall fire risk, an indicator contribution analysis is conducted. A perturbation-based approach is adopted, in which the score of each indicator is independently varied while all other indicators remain unchanged. This perturbation-based analysis evaluates the sensitivity of the composite fire risk index to variations in individual indicators, thereby enabling the identification of dominant risk drivers beyond the static importance reflected by AHP-derived weights.

A uniform perturbation of ±10% is applied to each indicator score to ensure comparability across indicators. The ± 10% perturbation level was selected following common sensitivity analysis practices in multi-criteria evaluation models, where moderate perturbations are introduced to examine the robustness of composite indicators without substantially altering the ordinal relationships among indicator scores. Additional tests using alternative perturbation levels (±5% and ±15%) indicate that the relative ranking of dominant indicators remains largely consistent.

The contribution degree of indicator i is quantified by the mean absolute variation of the comprehensive fire risk index, calculated as Equation (4).


$$C_{i}=\frac{\text{1}}{m}\sum_{j=\text{1}}^{m}\left|\overset{\left(i+\right)}{\underset{j}{R}}-\overset{\left(i\right)}{\underset{j}{R}}\right|$$ (4)

where *m* is the number of tunnel segments, $\overset{\left(i+\right)}{\underset{j}{R}}$ denotes the fire risk index of segment *j* after perturbation of indicator i, $\overset{\left(i\right)}{\underset{j}{R}}$ represents the corresponding risk index before perturbation.

### 2.6. Scenario-based risk analysis

To evaluate the effectiveness of different fire risk mitigation strategies, four representative scenarios were designed in addition to the baseline condition (S0). These scenarios simulate typical operational and management variations observed in cable tunnel systems. The adjustment ranges of the indicators in different scenarios were defined based on engineering operational practices and expert consultation in order to represent plausible variations in inspection intensity, protection system performance, and ventilation conditions within cable tunnel environments [25]. These scenarios are described as follows:

- S1: Inspection enhancement.

The indicator Inspection Frequency is adjusted from the baseline condition to the low-risk level (score = 1), representing more frequent and systematic inspection activities.

- S2: Protection improvement.

The indicators Fire Detector Reliability and Sprinkler System Reliability are improved by one risk level relative to the baseline condition (e.g., from medium risk to low risk), reflecting enhanced protection system performance.

- S3: Protection degradation.

The indicators Fire Detector Reliability and Sprinkler System Reliability are increased by one risk level compared with the baseline condition (e.g., from medium risk to high risk), representing deterioration or malfunction of fire protection facilities.

- S4: Ventilation improvement.

The indicator Ventilation Effectiveness is adjusted from the baseline condition to the low-risk level, reflecting improved ventilation conditions and enhanced smoke control capability.

For each scenario, only the specified indicators are adjusted, while all other indicators remain unchanged. The comprehensive fire risk index is subsequently recalculated using Equation (3) to evaluate the influence of different intervention strategies on tunnel fire risk.

### 2.7. Data processing and visualization

All data processing and calculations are implemented using Python. Indicator weighting, risk index calculation, contribution analysis, and scenario evaluation are performed programmatically to ensure reproducibility. Visualization techniques are used to present indicator weights, risk rankings, contribution degrees, and scenario comparison results.

### 2.8. Ethics statement

This study did not involve human participants, human specimens or tissue, vertebrate animals or cephalopods, vertebrate embryos or tissues, or field research. Therefore, ethical approval was not required for this work.

Prior to their participation in this study, all invited experts were fully informed of the research purpose, specific content to be evaluated, scoring criteria, research procedures, and the intended use of their scoring results. Verbal informed consent was obtained from each expert after they confirmed their understanding of the above information. The process of obtaining verbal consent was documented in detail in the research records, including the time of consent, the content communicated to the experts, and the experts’ confirmation of voluntary participation. Ethical review was not required for this study, as it only involved expert academic evaluation and scoring without any potential risks to the experts’ physical or mental health, personal privacy, or legitimate rights and interests.

## 3. Results

### 3.1. Indicator weighting results and contribution characteristics

**Fig 3:**
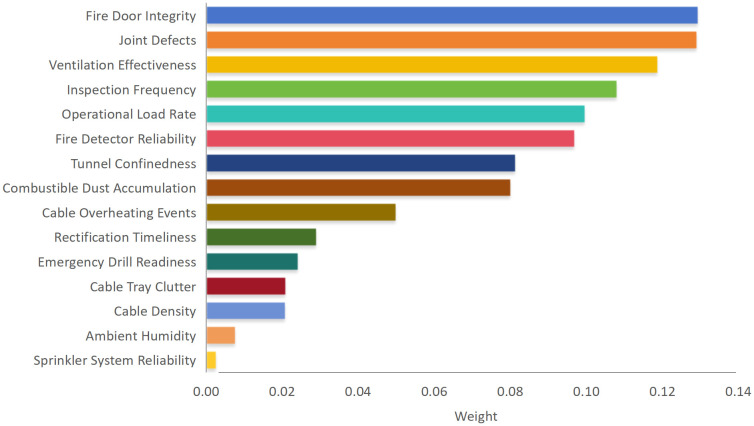
Fire risk indicator weights derived from AHP and their ranking.

The weighting results of the fire risk indicators obtained using AHP are shown in Fig 3, which illustrates the relative importance ranking of the indicators and highlights the uneven distribution of risk contributions across the indicator system. Indicator weights were derived from expert-based pairwise comparison matrices constructed using the standard nine-point AHP scale. All judgment matrices satisfied the consistency requirement (CR < 0.10), confirming the reliability and logical coherence of the weighting procedure.

The results reveal a markedly non-uniform distribution of indicator weights. Structural integrity related indicators, including Fire Door Integrity and Joint Defects, exhibit the highest weights among all considered factors within the studied cable tunnel system, together accounting for a substantial proportion of the overall fire risk. This suggests that deficiencies in compartmentation performance and structural continuity may play a dominant role in influencing fire risk formation within the studied cable tunnel system. A second tier of importance is formed by operational and management related indicators, such as Inspection Frequency, Ventilation Effectiveness, Operational Load Rate, and Fire Detector Reliability. These indicators are directly associated with ignition probability, smoke dispersion efficiency, and early fire detection capability, highlighting their important role in fire risk prevention and mitigation under routine operating conditions. In contrast, indicators related to environmental conditions, including Ambient Humidity and Cable Density, receive comparatively lower weights. This suggests that these factors primarily act as secondary modifiers of fire risk and exert a relatively limited influence on the overall risk level when considered independently.

**Fig 4:**
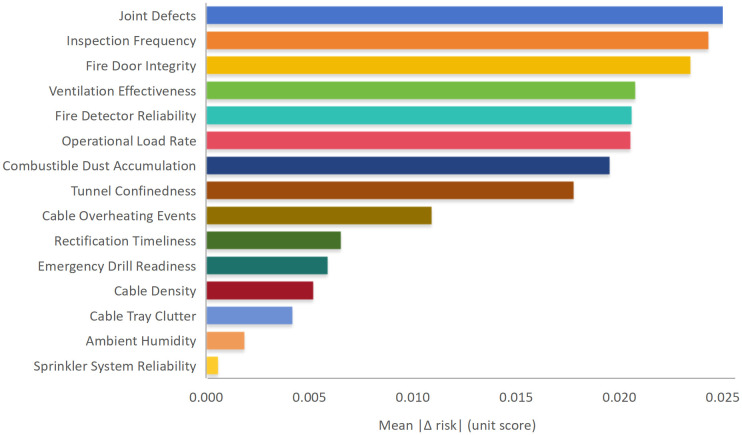
Sensitivity of the composite fire risk index to individual fire risk indicators.

To further evaluate the contribution characteristics of individual indicators, a contribution-oriented sensitivity analysis was conducted by perturbing each indicator score while holding all other indicators constant. Fig 4 presents the relative magnitude of the resulting variation in the composite fire risk index, highlighting the indicators with the strongest marginal influence on overall fire risk. The results indicate that Joint Defects, Inspection Frequency, and Fire Door Integrity produce the largest marginal impact on the overall fire risk index, which is consistent with their high weight rankings derived from AHP. This consistency between indicator weight magnitude and marginal contribution supports the robustness of the identified dominant fire risk drivers.

### 3.2. Segment-level fire risk assessment and spatial heterogeneity

**Fig 5:**
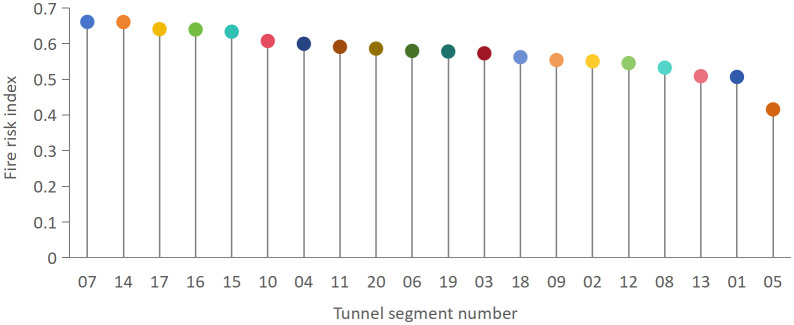
Ranking of tunnel segments based on composite fire risk index.

Based on the weighted aggregation of indicator scores, the fire risk index was calculated for each tunnel segment. In this study, a tunnel segment refers to a spatially defined section within the same cable tunnel system, delineated according to structural layout, operational conditions, and management characteristics. Each segment is assumed to exhibit relatively homogeneous fire risk attributes internally, while discernible differences may exist between adjacent segments due to variations in structural integrity, cable arrangement, operational load, and preventive management practices. The relative fire risk levels and ranking of the tunnel segments are illustrated in Fig 5.

The results reveal pronounced spatial heterogeneity in fire risk levels across the tunnel system. Several segments exhibit significantly higher fire risk indices, indicating the coexistence of unfavorable structural conditions, elevated operational load rate, and insufficient preventive management measures. These high-risk segments consistently show poor performance in indicators such as Joint Defects, Operational Load Rate, and Combustible Dust Accumulation, which collectively increase both ignition probability and fire escalation potential.

Conversely, segments with lower fire risk indices demonstrate more favorable inspection performance, improved compartment integrity, and reduced accumulation of combustible materials. This contrast highlights that fire risk is not uniformly distributed along the tunnel but instead concentrates in specific local sections where multiple adverse factors overlap.

The clear differentiation between high-risk and low-risk segments underscores the limitations of uniform safety management strategies applied at the tunnel-wide scale. Segment-level assessment enables the identification of localized risk concentrations that would otherwise be masked by aggregated evaluations. Consequently, the proposed segment-based fire risk assessment provides a quantitative basis for prioritizing inspection, maintenance, and retrofitting interventions, thereby supporting targeted risk control and more efficient allocation of safety management resources within cable tunnel systems.

### 3.3. Scenario-based risk variation and indicator interaction patterns

Four scenarios were selected to represent typical and practically actionable management and protection conditions encountered in cable tunnel operation. Specifically, inspection enhancement (S1) reflects intensified preventive maintenance strategies; protection improvement and degradation (S2 and S3) represent two contrasting states of fire protection system performance; and ventilation improvement (S4) corresponds to a commonly adopted engineering control measure. Together, these scenarios cover both management-oriented and engineering-oriented interventions, allowing the sensitivity of the composite fire risk index to representative control strategies to be examined.

**Fig 6:**
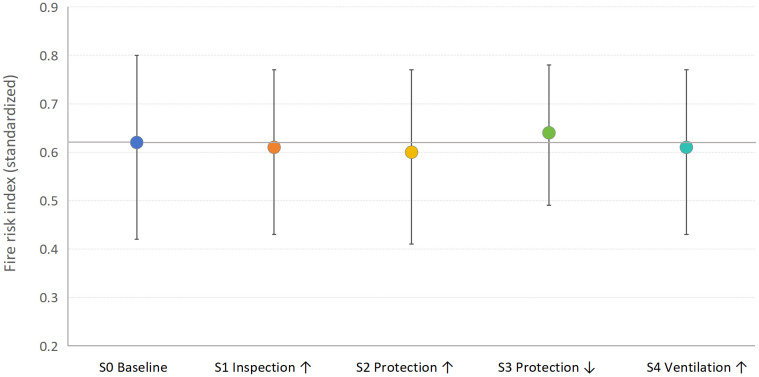
Comparison of composite fire risk indices under different operational scenarios (S0-S4).

To examine the response of the composite fire risk index to variations in management and protection conditions, multiple operational scenarios were analyzed. The comparative results of the composite fire risk index under different scenarios are illustrated in Fig 6, enabling an intuitive assessment of risk variation patterns induced by changes in key control measures.

Compared with the baseline scenario (S0), Scenario S1 shows a slightly lower mean fire risk index across tunnel segments. However, the distributions of the composite risk indices under different scenarios exhibit considerable overlap, suggesting that the differences among scenarios should be interpreted cautiously. Improvements in fire protection conditions (S2) are also associated with relatively lower risk index values across several tunnel segments, whereas degradation of fire protection systems (S3) tends to increase the composite fire risk index. Ventilation improvement (S4) shows a moderate influence on the risk index compared with inspection- and protection- related interventions. Overall, the scenario analysis provides indicative trends regarding the potential influence of different control measures on tunnel fire risk.

**Fig 7:**
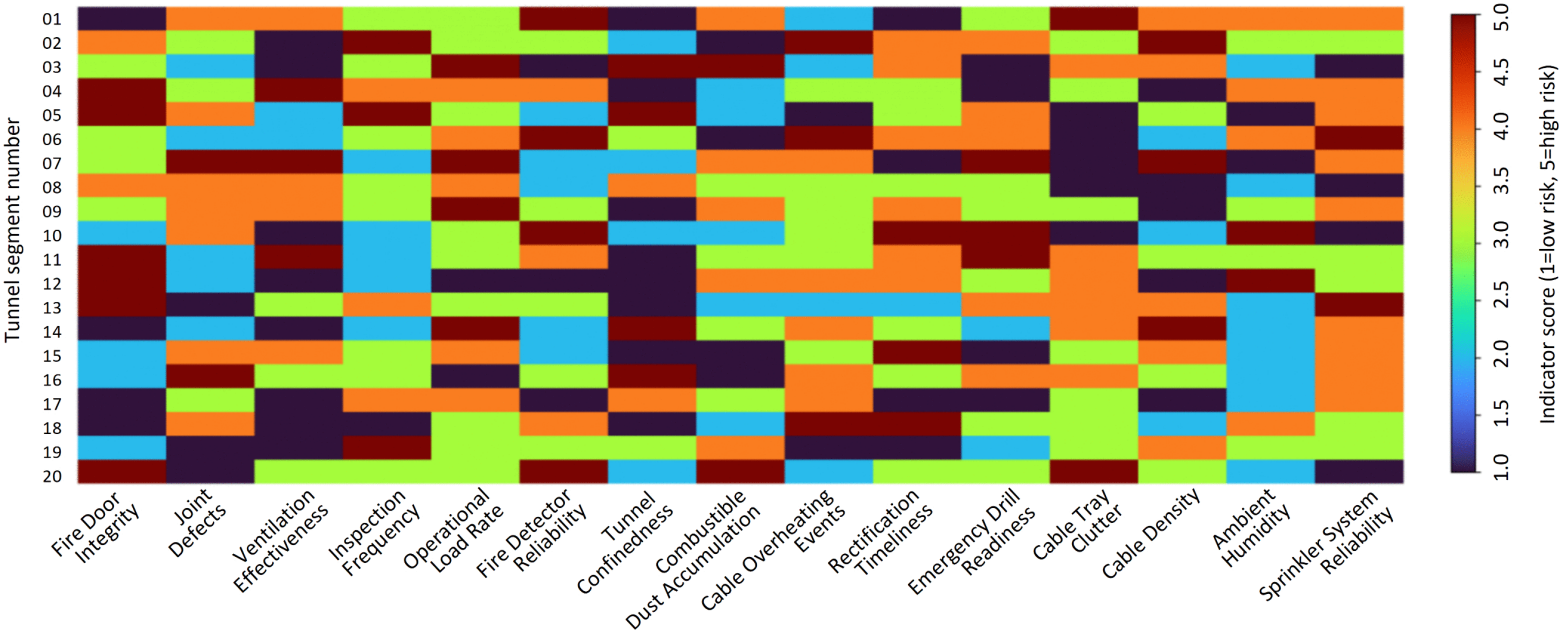
Heatmap of fire risk indicator scores across tunnel segments.

To further explore interaction patterns among indicators across tunnel segments, a heatmap representation of indicator scores is presented in Fig 7. Indicator scores are normalized using a unified five-level ordinal scale, where blue 1 indicates a favorable condition with minimal contribution to fire risk and red 5 represents a severely unfavorable condition with the highest risk contribution. The heatmap illustrates the co-occurrence patterns of adverse indicator states within high-risk tunnel segments. The results reveal that high-risk segments are typically characterized by the concurrent presence of unfavorable structural conditions, elevated operational loads, and insufficient management performance. This co-occurrence pattern indicates that fire risk escalation is not driven by isolated indicator anomalies, but rather by the combined and mutually reinforcing effects of multiple adverse factors acting simultaneously within the same tunnel segments.

## 4. Discussion

### 4.1. Dominant risk drivers and implications for tunnel fire safety management

The indicator weighting and sensitivity results from the studied cable tunnel system suggest that fire risk variation may be primarily influenced by a limited number of dominant factors rather than being evenly affected by all indicators. Structural integrity related indicators, particularly Fire Door Integrity and Joint Defects, consistently rank highest in both weight magnitude and marginal risk contribution. This finding underscores the critical role of compartmentation performance and structural continuity in restricting fire spread within cable tunnel environments.

In contrast, the relatively low weight associated with Sprinkler System Reliability in this study should be interpreted in the context of the specific characteristics of cable tunnel environments. In many cable tunnel systems, fire spread is strongly influenced by structural compartmentation and cable layout conditions [28,29], whereas active fire suppression systems often play a secondary role in early fire development stages. Consequently, structural integrity and operational management factors may exert a more significant influence on the composite risk index than sprinkler system conditions within the studied tunnel system.

From a process safety perspective, deficiencies in fire doors and structural joints directly compromise fire separation, allowing smoke and heat to propagate rapidly between tunnel sections. Unlike environmental factors that may fluctuate temporarily, structural defects often represent persistent latent hazards, which explains their disproportionate influence on the composite risk index. These results suggest that structural condition assessment should be prioritized as a foundational element of tunnel fire risk management.

Operational and management-related indicators, including Inspection Frequency, Ventilation Effectiveness, and Fire Detector Reliability, also exhibit substantial contributions to fire risk. Their influence reflects the importance of early hazard identification, effective smoke control, and timely fire detection in preventing escalation from incipient faults to full-scale fire events. The combined importance of structural and management-related factors highlights the necessity of integrating engineering integrity and operational discipline within tunnel safety strategies.

The findings of this study are generally consistent with previous research on fire risk assessment in underground infrastructures and utility tunnels. Earlier studies have emphasized the importance of structural conditions, combustible accumulation, and ventilation characteristics as key determinants of fire development and smoke propagation in confined tunnel environments. For instance, experimental and numerical investigations of cable tunnel fires have demonstrated that deficiencies in structural compartmentation and cable arrangement can significantly accelerate fire spread and increase thermal hazard levels [29,30].

Compared with existing indicator-based fire risk assessment approaches, the framework proposed in this study innovatively focuses on identifying dominant risk drivers through contribution-oriented analysis, which is a key improvement compared with existing indicator-based approaches that only focus on risk level ranking. This analytical perspective provides additional insight into how individual indicators influence composite risk formation, thereby supporting more targeted risk control strategies. In particular, the results highlight that structural integrity and inspection related management factors may play a disproportionately important role in determining tunnel fire risk, which is consistent with recent safety management studies on underground infrastructure systems [31,32].

Although expert-based weighting inevitably contains subjective elements, the consistency verification of AHP matrices and the subsequent perturbation-based contribution analysis together help enhance the robustness and interpretability of the proposed evaluation framework.

### 4.2. Spatial heterogeneity of fire risk and targeted risk control strategies

The segment-level risk assessment reveals pronounced spatial heterogeneity in fire risk across the tunnel system. High-risk segments are not randomly distributed but instead exhibit recurring patterns associated with unfavorable structural conditions, elevated operational load rate, and insufficient maintenance performance. This spatial concentration of risk has important implications for practical risk control.

Uniform safety management measures applied across all tunnel segments may lead to inefficient resource allocation and limited risk reduction effectiveness. Instead, the observed risk heterogeneity supports the adoption of differentiated management strategies, whereby inspection intensity, maintenance scheduling, and retrofitting priorities are adjusted according to segment-specific risk profiles. Such a targeted approach aligns with established principles of risk-based inspection and maintenance in process industries.

Moreover, the clear separation between high- and low-risk segments suggests that the proposed composite risk index can serve as a practical decision-support tool for prioritizing safety interventions. By identifying segments with disproportionately high risk contributions, tunnel operators can focus limited safety resources on locations where the potential safety gains are greatest.

### 4.3. Effectiveness of management interventions and multi-indicator interaction effects

The scenario-based analysis provides further insight into the effectiveness of different management and protection strategies. Enhancing inspection frequency and improving protection-related conditions both lead to measurable reductions in the composite fire risk index, confirming the value of proactive maintenance and protection reinforcement in mitigating tunnel fire risk. In contrast, the degradation of protection systems results in a pronounced increase in risk, emphasizing the vulnerability of tunnel safety performance to failures in protective infrastructure.

The relatively moderate risk reduction associated with ventilation improvement suggests that, while ventilation plays an important supporting role in smoke and heat control, its effectiveness is constrained when underlying structural or management deficiencies persist. This finding highlights the interdependent nature of tunnel fire safety measures and cautions against relying on single-factor interventions.

The heatmap-based interaction analysis further demonstrates that high fire risk levels typically arise from the concurrent deterioration of multiple indicators rather than from isolated deficiencies. Structural defects, high Operational Load Rate, and inadequate management performance frequently co-occur within the same tunnel segments, producing compounded risk effects. This interaction pattern reinforces the need for integrated risk management approaches that address multiple contributing factors simultaneously.

### 4.4. Practical implications and study limitations

The findings of this study offer practical guidance for cable tunnel fire risk management. Priority should be given to maintaining structural integrity, ensuring effective compartmentation, and sustaining robust inspection and maintenance regimes. The proposed indicator-based framework enables systematic identification of dominant risk drivers and high-risk segments, supporting evidence-based decision-making in tunnel safety management.

Several limitations should be acknowledged. The analysis relies on indicator scoring and weighting schemes that, although validated through consistency checks, inevitably involve expert judgment. In addition, the scenario analysis reflects predefined management changes and may not capture all operational conditions encountered in practice. Future research could incorporate real-time monitoring data or probabilistic fire simulation results to further enhance model fidelity and dynamic risk representation.

Overall, this study demonstrates that cable tunnel fire risk is a multi-factor, spatially heterogeneous phenomenon driven by the interaction of structural integrity, Operational Load Rate, and management effectiveness. The integration of indicator weighting, segment-level risk assessment, and scenario-based analysis provides a coherent framework for identifying dominant risk drivers, understanding spatial risk differentiation, and evaluating the effectiveness of targeted management interventions. These findings support the advancement of risk-informed fire safety management practices for cable tunnel systems and offer practical insights for improving safety performance in process-industry infrastructure. It should be noted that the findings are derived from a single cable tunnel case study. Therefore, the identified dominant risk drivers should be interpreted as case-specific observations rather than universal causal mechanisms. Future studies incorporating multiple tunnel systems or empirical fire incident data would further improve the generalizability of the proposed framework.

## 5. Conclusions

This study develops an indicator-based fire risk assessment framework for cable tunnel systems by integrating analytic hierarchy process weighting, segment-level evaluation, and scenario-based analysis. The results confirm that cable tunnel fire risk is driven by a limited set of dominant factors and exhibits pronounced spatial heterogeneity along tunnel segments.

In the studied cable tunnel system, structural integrity related indicators, especially fire door integrity and joint condition, appear to have relatively strong influence on the composite fire risk index, which may indicate the importance of effective compartmentation in restricting fire spread. Operational and management related factors, including inspection frequency and fire protection performance, may also contribute to variations in fire risk by influencing ignition likelihood and potential risk escalation pathways.

Segment-level assessment reveals that fire risk is spatially concentrated in specific tunnel sections where multiple adverse conditions co-occur, highlighting the limitations of uniform safety management strategies. Scenario-based analysis further demonstrates that proactive inspection and protection measures are effective in reducing fire risk, whereas deterioration of protective systems leads to rapid risk escalation.

Overall, the proposed framework provides a practical tool for identifying dominant fire risk drivers, prioritizing high-risk tunnel segments, and supporting risk-informed fire safety management in cable tunnel systems. Future research may incorporate real-time monitoring data or dynamic risk updating to further enhance applicability under complex operating conditions.

## Supporting information

S1 Appendix Complete AHP pairwise comparison matrices used for indicator weighting. (DOCX)

S2 Appendix Detailed qualitative scoring criteria for all fire risk indicators. (DOCX)
