# Supplementary material for: An Indicator contribution-oriented assessment framework for identifying dominant fire risk drivers in cable tunnels: Development and case study
Source: PLoS One. 2026 May 5;21(5):e0348198. doi: 10.1371/journal.pone.0348198 (PMC13143090; doi:10.1371/journal.pone.0348198)
Supplement: S1 Appendix — (DOCX) [file pone.0348198.s001.docx]

**Complete AHP pairwise comparison matrices used for indicator weighting**

The complete set of pairwise comparison matrices used in the analytic hierarchy process (AHP) weighting procedure are provided. The matrices were constructed based on expert evaluations using the standard nine-point AHP scale and aggregated using the geometric mean method.

**Table S1.** Pairwise comparison matrix for criterion-level indicators.

| Criterion | Structural Integrity | Environmental Characteristics | Equipment and Operational Conditions | Management Measures |
| --- | --- | --- | --- | --- |
| Structural Integrity | 1 | 3 | 5 | 7 |
| Environmental Characteristics | 1/3 | 1 | 3 | 5 |
| Equipment and Operational Conditions | 1/5 | 1/3 | 1 | 3 |
| Management Measures | 1/7 | 1/5 | 1/3 | 1 |

Note: This matrix represents the relative importance comparison among the four main criteria of the cable tunnel fire risk indicator system.

**Table S2.** Pairwise comparison matrix for structural integrity indicators.

| Indicator | Fire Door Integrity | Joint Defects |
| --- | --- | --- |
| Fire Door Integrity | 1 | 1 |
| Joint Defects | 1 | 1 |

Note: These indicators represent structural compartmentation performance and structural continuity within cable tunnel systems.

**Table S3.** Pairwise comparison matrix for equipment and operational condition indicators.

| Indicator | Operational Load Rate | Fire Detector Reliability | Sprinkler System Reliability | Cable Overheating Events | Tunnel Confinedness |
| --- | --- | --- | --- | --- | --- |
| Operational Load Rate | 1 | 2 | 3 | 3 | 4 |
| Fire Detector Reliability | 1/2 | 1 | 2 | 3 | 3 |
| Sprinkler System Reliability | 1/3 | 1/2 | 1 | 2 | 2 |
| Cable Overheating Events | 1/3 | 1/3 | 1/2 | 1 | 2 |
| Tunnel Confinedness | 1/4 | 1/3 | 1/2 | 1/2 | 1 |

Note:These indicators reflect operational conditions and fire protection system performance influencing ignition probability and early fire detection capability.

**Table S4.** Pairwise comparison matrix for environmental characteristic indicators.

| Indicator | Ventilation Effectiveness | Combustible Dust Accumulation | Cable Density | Ambient Humidity |
| --- | --- | --- | --- | --- |
| Ventilation Effectiveness | 1 | 3 | 4 | 5 |
| Combustible Dust Accumulation | 1/3 | 1 | 3 | 3 |
| Cable Density | 1/4 | 1/3 | 1 | 2 |
| Ambient Humidity | 1/5 | 1/3 | 1/2 | 1 |

Note: Environmental indicators describe surrounding physical conditions affecting fire spread and ignition probability.

**Table S5.** Pairwise comparison matrix for management measure indicators.

| Indicator | Inspection Frequency | Rectification Timeliness | Emergency Drill Readiness | Cable Tray Clutter |
| --- | --- | --- | --- | --- |
| Inspection Frequency | 1 | 3 | 4 | 5 |
| Rectification Timeliness | 1/3 | 1 | 2 | 3 |
| Emergency Drill Readiness | 1/4 | 1/2 | 1 | 2 |
| Cable Tray Clutter | 1/5 | 1/3 | 1/2 | 1 |

Note: Management-related indicators represent preventive management capacity and emergency response preparedness.
