# Supplementary material for: An Indicator contribution-oriented assessment framework for identifying dominant fire risk drivers in cable tunnels: Development and case study
Source: PLoS One. 2026 May 5;21(5):e0348198. doi: 10.1371/journal.pone.0348198 (PMC13143090; doi:10.1371/journal.pone.0348198)
Supplement: S2 Appendix — (DOCX) [file pone.0348198.s002.docx]

**Detailed qualitative scoring criteria for all fire risk indicators**

This supplementary material provides detailed qualitative scoring criteria for all fire risk indicators used in the cable tunnel fire risk assessment. All indicators are evaluated using a unified ordinal scale of 1-3-5, corresponding to low, medium, and high fire risk levels, respectively. Higher scores indicate higher fire risk.

**Table S6.** Scoring criteria for structural integrity related indicators.

| Indicator | Low Risk  (Score = 1) | Medium Risk  (Score = 3) | High Risk  (Score = 5) |
| --- | --- | --- | --- |
| Fire Door Integrity | Fire doors intact and fully functional | Fire doors partially degraded but still operational | Fire doors failed, missing, or ineffective |
| Joint Defects | No observable defects | Minor defects without structural discontinuity | Severe defects compromising compartmentation |

Note: Structural integrity indicators reflect the ability of tunnel structures to maintain fire compartmentation and limit fire spread.

**Table S7.** Scoring criteria for equipment and operational condition related indicators.

| Indicator | Low Risk  (Score = 1) | Medium Risk  (Score = 3) | High Risk  (Score = 5) |
| --- | --- | --- | --- |
| Operational Load Rate | Light operational load | High operational load | Overloaded condition |
| Fire Detector Reliability | Reliable and well-maintained | Degraded detection performance | Unreliable or malfunctioning |
| Sprinkler System Reliability | Reliable and fully functional | Degraded performance | Unreliable or non-functional |
| Cable Overheating Events | Rare or no overheating events | Occasional overheating events | Frequent overheating events |
| Tunnel Confinedness | Low confinedness with sufficient space | Moderate confinedness | Highly confined geometry restricting smoke and heat dissipation |

Note: Equipment and operational indicators are assessed based on operational records, monitoring data, and inspection results.

**Table S8.** Scoring criteria for environmental characteristic related indicators.

| Indicator | Low Risk  (Score = 1) | Medium Risk  (Score = 3) | High Risk  (Score = 5) |
| --- | --- | --- | --- |
| Ventilation Effectiveness | Favorable ventilation conditions | Restricted ventilation | Adverse ventilation conditions |
| Combustible Dust Accumulation | Minimal dust accumulation | Moderate dust accumulation | Severe dust accumulation |
| Cable Density | Low cable density | Moderate cable density | High cable density |
| Ambient Humidity | Suitable humidity conditions | Fluctuating humidity | Extreme or excessive humidity |

Note: Environmental indicators describe surrounding conditions that influence ignition probability and fire development.

**Table S9.** Scoring criteria for management measure related indicators.

| Indicator | Low Risk  (Score = 1) | Medium Risk  (Score = 3) | High Risk  (Score = 5) |
| --- | --- | --- | --- |
| Inspection Frequency | Regular inspection | Occasional inspection | Insufficient inspection |
| Rectification Timeliness | Timely rectification | Delayed rectification | Severely delayed rectification |
| Emergency Drill Readiness | Regular emergency drills | Occasional drills | Rare or no drills |
| Cable Tray Clutter | Organized cable trays | Moderate clutter | Severe clutter |

Note: Management-related indicators reflect preventive management capacity and emergency response preparedness.

**General Note:**

All indicators are evaluated using a unified ordinal scale of 1-3-5, where higher scores correspond to higher fire risk levels. The scoring criteria are fully consistent with the indicator system and weighting results presented in the main manuscript, and they support the comprehensive fire risk index calculation, indicator contribution analysis, and scenario-based evaluation.
